# Editorial for the Special Issue of Selected Papers from the 9th Symposium on Micro-Nano Science and Technology on Micromachines

**DOI:** 10.3390/mi10090618

**Published:** 2019-09-17

**Authors:** Norihisa Miki, Koji Miyazaki, Yuya Morimoto

**Affiliations:** 1 Department of Mechanical Engineering, Keio University, 3-14-1 Hiyoshi, Kohoku-ku, Yokohama, Kanagawa 223-8522, Japan; 2 Department of Mechanical and Control Engineering, Kyushu Institute of Technology, 1-1 Sensui-cho, Tobata-ku, Kitakyushu, Fukuoka 804-8550, Japan; miyazaki@mech.kyutech.ac.jp; 3 Department of Mechano-Informatics, Graduate School of Information, Science and Technology, The University of Tokyo, 7-3-1 Hongo, Bunkyo-ku, Tokyo 113-8656, Japan; y-morimo@hybrid.t.u-tokyo.ac.jp; 4 Institute of Industrial Science (IIS), The University of Tokyo, 4-6-1 Komaba, Meguro-ku, Tokyo 153-8505, Japan

The Micro-Nano Science and Technology Division of the JSME (Japan Society of Mechanical Engineers) promotes academic activities to pioneer novel research topics on microscopic mechanics. The division encourages interdisciplinary studies to deeply understand physical/chemical/biological phenomena at the micro/nano scale and to develop applied technologies. Since 2009, the past seven symposiums on Micro-Nano Science and Technology have taken place in a more interdisciplinary manner, incorporating the related societies of electronics and applied physics. We have promoted in-depth studies and interactions between researchers/engineers in various fields with more than 140 papers presented at each symposium for the past few years. Thanks to the previous activities and the great effort of the committee members, the Micro-Nano Science and Technology Division has been recognized as a formal division within the JSME.

This Special Issue collects 14 papers from the 9th Symposium on Micro-Nano Science and Technology, which was held from October 30 through 1 November 2018, in Sapporo, Hokkaido, Japan. All of the papers highlight new findings and technologies at micro/nano scales relating to a wide variety of fields of mechanical engineering, from fundamentals to applications. 

This issue present new fabrication technologies ranging from nano, micro, and mili scales. Direct writing of copper (Cu) in an ambient environment using femtosecond laser was proposed [1]. The laser reduces a glyoxylic acid Cu complex, which can be spin-coated onto a glass substrate. The resulting resistance of the patterned Cu was found to be large. The authors carefully investigated it and found the re-oxidation of the glyoxylic acid Cu complex to be the source. Nano-scale surface modification is known to be effective for control of heat transfer. Given the difficulty of direct observation of the phenomena, molecular dynamics simulation was conducted, which nicely explained the contact angle and water condensation at the surface [2]. Micro/nano fabrication is not limited to inorganic material but organic material that is soft, flexible, and biocompatible. Printing of a stimuli-responsive hydrogel, which includes printing an N-isopropylacrylamide-based stimuli-responsive pre-gel solution and an acrylamide-based non-responsive pre-gel solution in a supporting viscous liquid, and polymerizing the printed structures using ultraviolet (UV) light irradiation, was introduced [3]. Not only do the fabrication processes enable three-dimensional structures but the formed hydrogel can also respond to the stimuli. The authors claimed the process as 4D printing. Kirigami structures can generate large deformation with good controllability while the manufacturing process is rather two-dimensional and compatible with micro/nano technologies. However, the edges of the structures are typically not well constrained and cause instability in the motion. Therefore, a model comprising of connected springs in series with different rigidities in the regions close to the ends and the center is proposed [4]. It showed good agreement with experiments and will contribute to the theoretical design of kirigami structures.

Fabricated micro/nano features and devices must be assembled and packaged at the mili-scale to exhibit the best performance. The contact resistance when the electronic components are mounted using elastic adhesives was investigated, which is crucial in solderless writing in low temperature at low cost [5]. The careful investigation with respect to the contact pressure and Cu layer thickness led to the development of the sandwich structure to decrease the contact resistance. Micro/nano medical devices that exploit the small size and beneficial scale effects have been developed, however, the connection to the body is by far the most challenging. The connecting mechanism between the artificial blood vessels to facilitate the surgical procedure was proposed and demonstrated [6]. The mechanism allows blood to have contact only with the highly biocompatible surface; that is, the inner surface of the artificial blood vessels. The biocompatibility was experimentally investigated.

Sensors are one of the major applications of micro/nano technologies, which exploit beneficial scale effects in electro/magneto/mechanical science and engineering. A near-infrared spectrometer with a wide wavelength range using a plasmonic gold grating was proposed and demonstrated [7]. By improving the spectrum derivation procedure, the wavelength range covers 1200 to 1600 nm. A thin-film magnetic field sensor with a logarithmic amplifier was newly proposed [8]. The amplifier can translate hundreds of MHz signals to a direct current (DC) voltage signal which is proportional to the radio frequency (RF) signal. A whole sensor system can be small enough to be practically used to detect foreign materials in industrial and medical products. Tactile sensation is considered to be the next tool for the intuitive and efficient human/computer interface. A thermal tactile sensation display, which controls the effective thermal conductivity, was proposed and demonstrated [9]. A highly thermally conductive liquid metal is introduced into the device, whose amount controls the effective thermal conductivity of the device. The range of the effective thermal conductivity was experimentally deduced and human perception tests were conducted to verify the concept.

Micro/Nano fluidics have been studied from their fundamentals to their biomedical applications. This Special Issue covers these topics with five papers. First, separation of nano- and micro-particle flows in branched microfluidic channels using thermophoresis [10]. Localized temperature increases near the branch are achieved using the Joule heat from a thin-film micro electrode embedded in the bottom wall of the microfluidic channel. The particle flow into one of the outlets is blocked by microscale thermophoresis since the particles are repelled from the hot region in the experimental conditions used here. The nano-particle case was also discussed theoretically and experimentally. The steady streaming that can generate net mass flow from zero-mean vibration is attracting many researchers in this field. To achieve the steady streaming, the numerical analysis for three-dimensional and unsteady flow was proposed [11]. The particle trajectories induced around a cylindrical micro-pillar under circular vibration was solved in the Lagrangian frame and the results were converted to a stationary Eulerian frame to compare with the experimental results, which showed good agreement. The proposed model can be a strong tool to design the micro scale flow of interest.

Biomedical applications using micro/nano fluidics and biocompatible polymer material, in particular hydrogels, are discussed. The degeneration of adipocyte has been reported to cause obesity, metabolic syndrome, and other diseases. To treat these diseases, an effective in vitro evaluation and drug-screening system for adipocyte culture is required. An in vitro three-dimensional cell culture system to enable the monitoring of lipid accumulation by measuring electrical impedance was proposed [12]. The relationship between the impedance and lipid accumulation of adipocytes was investigated experimentally and the lipid accumulation of adipocytes was found to be monitored in real time by the electrical impedance during in vitro culture. Reconstructing a three-dimensional muscle using living cells is promising for restoration of damaged muscles. However, the regenerated tissue exhibits a weak construction force due to the insufficient tissue maturation. A cell-laden core-shell hydrogel microfiber as a three-dimensional culture to control the cellular orientation with cyclic mechanical stimulation was proposed and demonstrated [13]. The directions of the myotubes were oriented and the mature myotubes could be successfully formed by cyclic stretch stimulation. An anchoring device with pillars to immobilize an adipocyte microfiber was proposed to track the specific positions of the microfiber for a long period [14]. Temporal observations of the microfiber on the device for a month successfully revealed the function and morphology of three-dimensional cultured adipocytes. Lipolysis of the microfiber’s adipocytes by applying reagents with an anti-obesity effect was also demonstrated, which indicates the effectiveness of the system for drug tests.

We would like to thank all the contributing authors for their excellent research work. We appreciate all the reviewers who provided valuable comments to improve the quality of the papers and the tremendous support from the editorial staff of Micromachines.
